# Tin: A Plea for More Conservative Methods in Filling Teeth

**Published:** 1901-07

**Authors:** T. D. Shumway

**Affiliations:** Plymouth, Mass.


### TIN: A PLEA FOR MORE CONSERVATIVE METHODS IN FILLING TEETH.[1]

BY T. D. SHUMWAY, PLYMOUTH, MASS.

In submitting this subject for your consideration we wish to disclaim any intent to show superior manipulative ability in the use of cohesive gold, or to merely demonstrate by a novel experiment the possibility of uniting gold with tin by simple contact. If this were the only reason for appearing before you, it would be well not to consume valuable time which could be more pleasantly and profitably employed. I trust, in this Society, the day is gone by when exhibitions of this character continue to excite either wonder or admiration. That there have been demonstrations of this sort far beyond the mechanical skill of your essayist to excel, or even to emulate, no one is more willing to concede.

It is for the purpose of presenting the hackneyed subject of filling teeth in a somewhat different manner from that in which it is usually treated that the above title was selected and these specimens of the work submitted for your inspection. These specimens

---

[1] Read before the Harvard Odontological Society, December 27, 1900. Copyright, 1901.

are not intended to be like those made with cohesive gold and the mallet. In their physical construction they are radically different. Whatever value they possess consists in this fact.

Had the use of cohesive gold and the mallet met the expectations of the profession, it would be presumptuous, indeed, to attempt to offer anything in its place. It is true, the skill displayed in the repair of wasted and broken tissue, by contouring, has been something remarkable. Manual dexterity has reached a high state of development, but the results obtained have been at a fearful sacrifice of tooth-structure. History repeats itself, and the same objections which caused the failure of crystal or sponge gold, nearly fifty years ago, applies to any form of cohesive gold when placed in contact with tooth-substance by mallet force. In the discussions relating to the use of crystal or sponge gold, it was pointed out what would follow this plain violation of natural law, in impaired or arrested function. In an article by Dr. J. De Haven White, published in the *Dental News Letter* of July, 1854, the writer says, "It is believed by some that a plug must be impervious to dampness; this cannot be, or, if it were, it would not be necessarily a perfect plug; dampness must permeate a plug to some extent, or the dampness will force around the plug and displace it sooner or later. We know well that a distinguished operator in our city loses more hard plugs than soft ones on that account; his plugs are therefore better than the teeth he puts them in. We do not wish to be understood as advocating hard plugs, but we believe the most perfect plug is of about equal porosity to the dentine; with a good cavity it will remain in longer than a harder plug, especially in the lateral portion of the teeth. A foil plug will not be broken up by such permeability, and a sponge plug will. No reasonably good operator loses a plug by softening, but by the margins of his cavity giving way. The constant expansion and contraction of the plug and the tooth will cause any plug to give way sooner or later, and, until we get a substance that will expand and contract with the tooth, so as not to loosen its margins, we will have some of our highest specimens of art crumbling away under our eyes."

It is evident that Dr. White had an intelligent understanding of structure and function. As a student of vital energy he was able to discern the cause of failure in the use of crystal or sponge gold. Manipulating cohesive gold by the more modern methods does not remove the objections which he so forcibly stated. The introduction of the rubber dam made possible operations which before were only partially successful, but the evil was augmented in a more thorough crystallization of the gold by mallet force.

That many teeth have been filled with cohesive gold and the mallet which have remained for a great number of years, no one will attempt to deny. There are many people that have survived and enjoyed a comfortable degree of health who have violated the laws of correct living. There is a wonderful recuperative energy in the human organism. But for this it is a question if the race would not long since have become extinct by reason of transgression of natural law. Admitting that some teeth which have been filled with cohesive gold and the mallet are doing good service, we believe this is due to the recuperative energy that was able to overcome the contact of a foreign body incompatible with tooth-structure, rather than the influence of the filling itself. With the record of failure, it is fair to say the success of filling teeth with cohesive gold has not been commensurate with the amount of labor bestowed, the physical exhaustion, mental strain, and nervous tension on the part of the operator, together with the pain and suffering the patient has been made to endure. It is significant that crowns and bridges and inlays should follow so closely in the track of cohesive gold and the mallet. It is also significant that those who became the most expert in using gold in this form were early in the field to adopt the later method of cutting off crowns.

When the mallet was introduced for the purpose of condensing cohesive gold, it was assumed that, because lost tissue could be restored, the teeth would be preserved by purely mechanical means. Operative dentistry, or the care of the natural teeth, like the practice of medicine, is not an exact science, although it has to deal with scientific subjects. Nature rebels at any attempt to reduce her methods to exact mathematical lines. This is clearly demonstrated by a study of structure and function in tooth development. This study has to be prosecuted under difficulty, for no one is privileged to see the secret workings of nature. The microscope can only reveal what has taken place, but not what is going on in this workshop. If we could examine, under the lens, without first destroying the vital force, what secrets would be unfolded! It is by reasoning from what we know, that we are able to reach conclusions which are to guide us.

In the discussion of this subject the first inquiry, then, is, How is a tooth developed, and what are the changes wrought in this wonderful organ from its beginning to old age? or, in other words, How is a tooth builded? At its inception there is the dental arch, in which appears a groove, and across this groove, which divides it into pockets, there shoots a thin porous bone. In the pockets are follicles, which are the germs or buds of the future teeth. These follicles are connected with the circulatory system by arteries from the internal maxillary branch of the external carotid, and veins from the internal maxillary vein, which returns the blood, and terminates in the external jugular, and nerves from the fifth cerebral or fifth pair. Here we see that every pulsation of the heart sends forth the material for tooth formation. Embryologists tell us that at the end of the fifth or the beginning of the sixth month of fœtal life the process of enamel formation is about to commence. The cells which form the external epithelium, or Nasmyth's membrane, have performed their function and disappeared. This membrane has a polished surface, and is a covering for the prisms or rods which are to form underneath. These prisms or rods are held together by what is termed a cementing plasm.

In the formation of enamel is first seen the form of the future tooth in a cutting edge for an incisor and a cusp for a bicuspid or molar. The enamel-organ when completed is a purely crystalline formation, and, according to the best authorities, is without any trace of organic matter. All crystalline bodies are formed from without inward, and enamel formations can be no exception to this rule. A little reflection will make this apparent. Take, for example, the shell of a lobster or the skin of a snake. When a lobster sheds its shell, it is provided with a membrane similar to the outside covering of enamel of the teeth, which is highly polished, but extremely tough and flexible. In the process of time there is formed underneath and attached to this membrane a calcareous deposit, crystalline in character, varying in thickness from one-sixteenth to one-fourth of an inch. We can readily see how impossible it would be for the lobster to shed its shell but for this crystalline process taking place from without in. Nature provided an inorganic covering which acts as a protection during the process of calcareous formation. Suppose the process to be reversed. It would simply mean the death of the lobster. After the new shell is formed, another membrane appears, and in due time the old shell is thrown off, and the lobster comes forth increased in size. In no other way could the lobster get out of its environment and expand and grow.

What a beautiful sight is a black-snake that has just shed its skin,—so black and glossy! Now, this new skin is pure crystal, and is formed in the same manner as the lobster's shell, only differentiated to meet the habitat of the snake. Nature had to provide an inorganic covering to prevent the snake from being torn and bruised by the stones and bushes over and through which it crawls.

On a cold winter's day watch the process of crystallization on the window-pane. It is always from the sides towards the centre, and never from the centre outward. This is the key that opens the secret chamber of nature and reveals the process of enamel formation for the teeth. When once formed, the enamel plays no part in the life of the organ, any more than the lobster's shell or the snake's skin, except to act as a protecting covering. The enamel of a tooth is the only part of the animal economy which does not undergo decomposition or change. Professor R. R. Andrews, in a paper read before the recent International Dental Congress, says, "The finest lenses do not reveal the slightest differences between enamel ground from a living tooth and that which has laid in the ground for centuries."

The growth of the dentine is by a process the reverse of that by which the enamel is formed; that is, its growth is from within out. Unlike enamel, it is composed of two distinct parts, minute tubes and a fibrous tissue called the uniting medium. In their arrangement these tubes radiate from a centre. Tomes says, "The centre of radiation is the pulp-cavity." These tubes ramify in undulations not only towards the enamel, but also dip downward to form the root of the tooth. They also extend through the sides of the root, and are lost in the cementum.

This fact should have an important bearing in the treatment of pulpless teeth. As yet, we have only the framework of a tooth. It is like a house under the process of construction, with an outside covering and partition walls. In the work of tooth-building these tubes perform a very important function. They are filled with a substance that is given off from the pulp, which, so far as the microscope can determine, is amorphous or structureless. This amorphous substance, granular in character, changes into a lime-salt. This change is going on from the time the tooth is formed until the period of extreme old age, when the pulp almost entirely disappears. There is no part of the human organism where the process of change is carried on more continuously than that which takes place within the teeth. Although the teeth are provided with an indestructible covering, they are victims of disease.

From some cause, which it is not necessary to inquire into in this connection, the enamel becomes abraded, and the dentine is attacked with caries. Nature has provided for attacks of this kind by making the point where the amorphous substance is changing into a lime-salt the most sensitive part of the organ. She at once sets up the work of repair by a secondary deposit of dentine. The sensation of pain which is experienced is not only a note of warning, but a cry for help. This cry is for a protecting covering. If the waste is greater than the repair, it means destruction of the organ. The more recent investigations have shown that the work of repair goes on even after the pulp has become inflamed.

In the *Dental Cosmos* for November is published a paper read before the International Congress at Paris, by Dr. A. Hopewell Smith, of London, on "Certain Adventitious Dentine associated with Inflammatory Conditions of the Dental Pulp," in which he says that "in the majority of the inflammations, but by no means in every inflammatory condition of the dental pulp, there is found a protecting layer of hard adventitious dentine, which is put in the place of danger; that is to say, opposite the breach by caries of the surface."

Here we see the effort of nature to protect herself even after the struggle has become hopeless. From this it would seem that operations upon the teeth, to stay the inroads of caries, should be protective and remedial. The dentist cannot save teeth any more than the practitioner of medicine can cure disease. Nature must work the cure. The aim of operative dentistry should be, primarily, to assist nature, not a display of mechanical skill, in disregard to fundamental law.

A study of the material to place in contact with tooth-substance is the first requisite, if we would approach the operation scientifically. A crystalline formation is obstructive when placed in contact with a living or fibrous body. No one would expect to ripen apples by filling them with gravel stones. If you should shoot malleted fillings of cohesive gold into the body, you would invite blood-poisoning. The same law governs in the more dense substance of the dentine, which is filled with life. Again, it should not be anchored with dovetails, angles, retaining-pits, etc., as nature is above mathematics, and refuses to be bound. This is the reason that Dr. White says "the filling should expand and contract with the tooth," to meet the changing conditions. An amorphous substance which is undergoing a metamorphosis demands that the material to be placed in contact with it shall be as structureless as the substance itself.

An illustration of this is seen in the way lead or tin becomes encysted when buried in the soft tissue. Of all the materials that have been employed as a tooth-stopping, there are none which have shown such preservative properties as tin. It has a history and a record as old as operative dentistry. In 1825 Mr. Sigmond, of Bath, England, said, "In 1783 I stopped a considerable decay in a large double under tooth, on the outside of the crown, or near the gums, with fine tin-foil, which lasted for a good number of years."

The saving properties which tin exhibits when placed in a carious tooth are well established. The testimony of many skilful dentists bears witness to this fact. The reasons that have been given as to why tin exhibits this property have been numerous and varied. Some have attributed it to a therapeutic property in the metal; others, like Dr. Palmer, of Syracuse, that it is positive to electrochemical action, and some, like Dr. Miller, of Berlin, that it is germicidal. When compared with other metals, tin may be said to be almost amorphous. Its specific gravity is 729; it melts at 442° F.,—a little more than twice the boiling-point of water. Gold is more than four times as tenacious, and six times as good a conductor of heat. Under the same conditions, tin expands nearly twice as much as gold, but the rate of expansion of gold is nearly twice as much as tin. Here is a metal that, in its physical characteristics, borders upon the structureless. When tin is placed in contact with the amorphous substance of the dentinal tubuli, it acts as an assistant in the effort of nature to protect herself from untoward external conditions. This, it would seem, is the more scientific reason for its benign influence.

The limitations of tin have been the barrier to its general use. These limitations are its color, softness, and the tendency to what is termed oxidation. Various methods have been suggested to meet these objections, one of them being to make a cylinder of tin- and gold-foil, and then force this into place, either by the wedging process or by the mallet. But this method has only been partially successful. Incorporating gold with the tin destroyed the attractive appearance of the gold, and the tin would oxidize and wash out where it came to the surface.

The idea of covering tin with gold is not new. It was practised nearly fifty years ago. The method was to force the gold into a base of tin with a sharp instrument, with the object to simply cheapen the operation. This was the only value the author claimed for this method. In the *Dental News Letter* of January, 1856, in a communication by Dr. F. Y. Clark, on "Material used in filling Teeth," the writer says, "Now, as gold is too costly, and tin is too soft, what are we to do with those poor patients that are continually seeking our aid? The plan that I adopted two or three years ago I find as yet works well. At first I had many misgivings on account of two metals being placed in contact in the same tooth, but I can see no difference in placing two metals in contact in a tooth than in placing them in contact around it. But I think the evil is more imaginary than real, for so far I have not been able to detect the least trouble. Therefore, when I have a patient who is not able to pay me the worth of the gold used (I speak of teeth with large cavities on their grinding surface), I commence with tin and fill up the fangs (if I have removed the nerve), also the pulp-cavity, and as much of the crown as I safely can. I pack the tin in perfectly hard, leaving or making it flush, and then, with annealed number four gold-foil, I finish off. I use number four because I find it can be forced into the little threads in the walls of the cavity better than any other number when annealed. Now, I cannot see why such a filling is not just as good as if it were all gold. The tin cannot wear nor corrode, for it has not the slightest chance, and the gold on the surface is hard enough to resist all antagonists of the mouth; and, as the cost is not more than one-third as much as it would be if it were all gold, we can get pay for the gold, if not for our work. I always adopt this plan when I have to fill a tooth on the grinding surface, from which I have taken the nerve, for less than six dollars. In the incisor teeth tin, or any other metal but gold, should not, of course, be used. All cavities that can be filled with tin can be filled equally well with gold; therefore, we have no inducement to use tin but its cheapness."

It evidently did not occur to Dr. Clark that he might possibly be giving a better service, for smaller compensation, to his poorer than to his more fortunate patient who was able to pay for all gold. The method he used was solely for the purpose of economy. The idea that a filling must be in harmony with its environment, and not obstruct, but aid, nature in her work of repair, formed no part of his method, as the object was to get something cheap.

The value of a filling depends upon its ability to save teeth, without regard to the cost of the material used. The only other consideration is that it shall offend the eye as little as possible. If the theory that tin exercises a benign or healing influence when placed in a carious tooth be sound, the problem is how to eliminate its objectionable features and extend the boundary of its usefulness. A cavity filled with tin, with an outside or protecting covering of cohesive gold, laminated, so as not to incorporate the gold in the tin, is in correspondence with nature, and more fully meets the demand of its surroundings.

Much that has been said in condemnation of tin as a filling-material is proved, on examination, to be without good reason. That tin-foil, when made from the pure metal, disintegrates, or becomes powdery, is shown to be false. It is a mistake to charge tin with making the teeth black, when, really, it makes them of a brighter hue. Remove a tin filling after it has been in the cavity of a tooth for years, and the dentine will present a bright, healthy, dense appearance. Tin never penetrates the tubuli like amalgam.

But it may be urged that all this sheds no light upon the most important part of this subject,—*i.e.*, the practical application, or how to fill the teeth with tin so as to remove the objections and retain its virtues. In the filling of teeth the first step is the preparation of the cavity, and in the use of tin there are no exceptions to the general principles laid down in the various text-books. Unsupported walls of enamel are to be broken down and the decayed dentine removed. In doing this, however, no more tooth-structure need be sacrificed than is necessary to accomplish the result. Being interdigitous, it spreads laterally, and does not need angles, dovetails, undercuts, or retaining-pits to hold it in place. It is not refractory, like cohesive gold, which only stays when it is anchored, so that it cannot get away, but yields readily to pressure. It remains in place not from necessity, but from choice. The removal of healthy tooth-structure for retention is not required. It does not call for "extension for prevention," as tin itself prevents extension. By reason of this saving of tooth-structure many fillings may be almost entirely concealed.

Tin is introduced and manipulated in the same manner as has been so often described in making cylinder fillings by the wedging process. It should be condensed into a solid mass, so that it may be cut with a sharp chisel or excavator. This is best accomplished by heating the plugger in an alcohol or gas flame to a degree to render the tin malleable. This requires about 212° F. The sensation of heat which the patient experiences is not so acute as that which follows the introduction of hot gutta-percha, provided it is done with judgment and care. When the tin is consolidated, it should be flush with the enamel walls. So far the work is done with the old-fashioned hand-pluggers with large handles. The next step is to bring the flattened surface of a very light and extremely cohesive gold cylinder in contact with the surface of the tin, when a union of the metals takes place at an insensible distance, like the uniting of two drops of water. Continue this process until there is an outside covering of gold, which will finish down to a smooth and polished surface. For finishing, all that are required are sand-paper, cuttle-fish disks, and strips. It does not need to be burnished, as burnishing tends to impair the union of the metals by drawing the gold away from the margins.

All crystalline bodies under force or pressure assume a definite form. The crystals of gold being spheroidal, the tendency under pressure is always towards the centre. The same law which governs in the vital organ should be recognized in the treatment of pulpless teeth. It is a misnomer to call a pulpless tooth a "dead tooth." It is true that the process of tooth-building is stopped with the death of the pulp, but as the tubuli extend through the sides of the root, a certain amount of vitality may be maintained under proper treatment. This treatment should be in a way to cause as little change as possible in the conditions under which the tooth was found.

There can be no question that tin in the root-canal is as bland as it would be if encysted in the muscular tissue. Clinical experience has demonstrated that a root properly filled with tin will remain perfectly odorless, while one filled in the same manner with cotton and cement, or gutta-percha, almost invariably gives off a most offensive odor on being removed. This odor is of the kind called "brassy," and is, no doubt, largely due to a decomposition of the amorphous substance which has been arrested in its metamorphosis. A similar odor is often detected on removing a cohesive gold filling placed in a living tooth, which had been inserted with great care.

The question which naturally suggests itself is, Will a filling of this kind stand the test of crushing stress equal to those of cohesive gold or amalgam? Dr. White says "the best filling is one of about equal porosity with the dentine." Hardness, or the power to resist force when applied out of the mouth is not a scientific way of testing the value of a filling when placed in a tooth, as the conditions are in no way similar. As before stated, the object is to obtain a filling that in its physical construction shall as nearly as possible conform to the healthy organ. The teeth are not set in the sockets the same as a post is put in the ground. They are cushioned in the jaw, and give way under pressure. Besides, the material of which they are made is the most elastic of any known substance. For this reason, billiard balls are always made of ivory.

There have been many teeth filled with tin on the grinding surface that have worn out in the centre, and yet protected the walls of the cavity sufficiently to permit nature to make a secondary deposit, almost as dense and hard as the enamel with which it was originally covered. A secondary deposit of dentine, under a malleted cohesive gold filling would be rare, indeed. How often do we see teeth crumble away while these fillings remain intact. It is more scientific to have the fillings wear and save the teeth than to have the fillings remain and the teeth decay. In one case, the filling may be easily replaced; in the other the organ is lost, while the work remains.

This is not intended to cast any doubt upon the ability of the fillings we suggest of withstanding the force of ordinary attrition when placed in any part of the mouth which has been affected with caries. A practical application of this method in many different positions has convinced your essayist of the correctness of the principle that correspondence and harmony, and not "crushing stress," are the true factors in tooth preservation.

The points we have endeavored to enforce are, first, that the enamel of a tooth, being formed from without in, plays no part in tooth development or preservation, except to act as a protecting covering; that the process of tooth-building is from within out, and continues from infancy to old age; that nature attempts to protect herself from the encroachment of disease by a deposit of secondary or adventitious dentine; that if the cause of decay is external, the recuperative force is from within; that the operation of filling teeth should be based upon a recognition of these processes of nature, and that the material when placed in contact with the dentine should be in correspondence with the amorphous or structureless substance with which the tubes are filled; that of all the materials which have been employed, tin most nearly meets these conditions; that the objections to tin of color, softness, and a tendency to oxidation are met by proper manipulation, and the field of its usefulness extended; that it suggests a theory of practice scientifically correct.

If these conclusions are well founded, it removes from the operation of filling teeth the empirical and the doubtful, and inspires the patient with confidence and hope. Being in harmony with natural law, it lifts a burden from the shoulders of the practitioner, and makes a pleasant duty of an irksome task. Having a scientific basis for its foundation, it elevates the standard of dentistry and lends dignity to a worthy calling. Above all, it mitigates the pain and suffering attendant upon operations in the mouth, from which even the most heroic shrink. If we succeed in dispelling the doubt of its practical utility from a single mind, our labor will not be in vain.

At least, it is not unreasonable to expect that in the presentation to this Society of a subject so important, both to dentist and to patient, it will stimulate discussion and evoke criticism. To this field we invite you, as no one mind can be the repository of all knowledge.

